# Case Reports: Novel Missense Variants in the Filamin C Actin Binding Domain Cause Variable Phenotypes

**DOI:** 10.3389/fneur.2022.930039

**Published:** 2022-07-12

**Authors:** Daniele Velardo, Maria Grazia D'Angelo, Andrea Citterio, Elena Panzeri, Laura Napoli, Claudia Cinnante, Maurizio Moggio, Giacomo Pietro Comi, Dario Ronchi, Maria Teresa Bassi

**Affiliations:** ^1^Fondazione IRCCS Ca' Granda Ospedale Maggiore Policlinico, Neuromuscular and Rare Diseases Unit, Department of Neuroscience, Milan, Italy; ^2^NeuroMuscular Unit, Scientific Institute for Research, Hospitalization and Healthcare (IRCCS) E. Medea, Bosisio Parini, Italy; ^3^Laboratory of Molecular Biology, Scientific Institute for Research, Hospitalization and Healthcare (IRCCS) E. Medea, Bosisio Parini, Italy; ^4^Fondazione IRCCS Ca' Granda Ospedale Maggiore Policlinico, Neuroradiology Unit, Milan, Italy; ^5^Department of Radiology, Istituto Auxologico Italiano, Scientific Institute for Research, Hospitalization and Healthcare (IRCCS), Milan, Italy; ^6^Department of Pathophysiology and Transplantation, Dino Ferrari Center, Neuroscience Section, University of Milan, Milan, Italy

**Keywords:** Filamin C, actin binding domain, distal myopathy, muscle electron microscopy, muscle magnetic resonance imaging, next generation sequencing

## Abstract

Filamin C is a large dimeric actin-binding protein, most prevalent in skeletal and cardiac muscle Z-discs, where it participates in sarcomere mechanical stabilization and intracellular signaling, interacting with numerous binding partners. Dominant heterozygous mutations of Filamin C gene cause several forms of myopathy and structural or arrhythmogenic cardiomyopathy. In this report we describe clinical and molecular findings of two Italian patients, in whom we identified two novel missense variants located within the Filamin C actin binding domain. Muscle imaging, histological and ultrastructural findings are also reported. Our results underline the extreme inter- and intrafamilial variability of clinical manifestations, hence the need to extend the investigation also to asymptomatic relatives, and the relevance of a broad diagnostic approach involving muscle electron microscopy, skeletal muscle magnetic resonance imaging and next generation sequencing techniques.

## Introduction

Heterozygous defects in the human Filamin C gene (*FLNC*) located on chromosome 7q32.1 result in clinical forms of myopathy and cardiomyopathy with marked phenotypic variability (1, 2). FLNC-related myopathies comprise three main presentations, according to type and location of the molecular defect: (i) missense or splice site changes affecting the rod domain result in late onset, progressive, proximal muscular weakness with large sarcoplasmic inclusions; (ii) frameshift mutations in the rod domain cause distal myopathy without sarcoplasmic inclusions; (iii) missense variants in the actin-binding domain (ABD) result in proximal or distal myopathy with non-specific myopathic changes (3–5). More recently, patients displaying restrictive, hypertrophic, dilated and arrhythmogenic cardiomyopathies have been found harboring truncating and missense *FLNC* mutations (6). Here we describe two novel *FLNC* variants located in the actin-binding domain associated with different phenotypes in two distinct Italian families.

## Case Descriptions

Samples collection and studies were performed with informed consent from the patients according to approvals given by Ethic Committee of the Scientific Institute IRCCS Eugenio Medea, Bosisio Parini, Lecco, Italy and IRCCS Ca' Granda Foundation, Ospedale Maggiore Policlinico, Milan, Italy. The complete timeline of relevant clinical signs and symptoms and of diagnostic assessments performed during disease progression is reported in Supplementary Figure 1.

### Patient 1

Patient 1 is a 53-year-old man, born to non-consanguineous parents, who had noticed mild hand and triceps brachii muscles wasting without corresponding weakness at the age of 28. Before then, he had practiced sport at a competitive level. At the age of 31 he started to complain fatigue and weakness in the legs and arms, impairing daily activities. Muscle cramps were present, mostly in the lower limbs.

**Figure 1:**
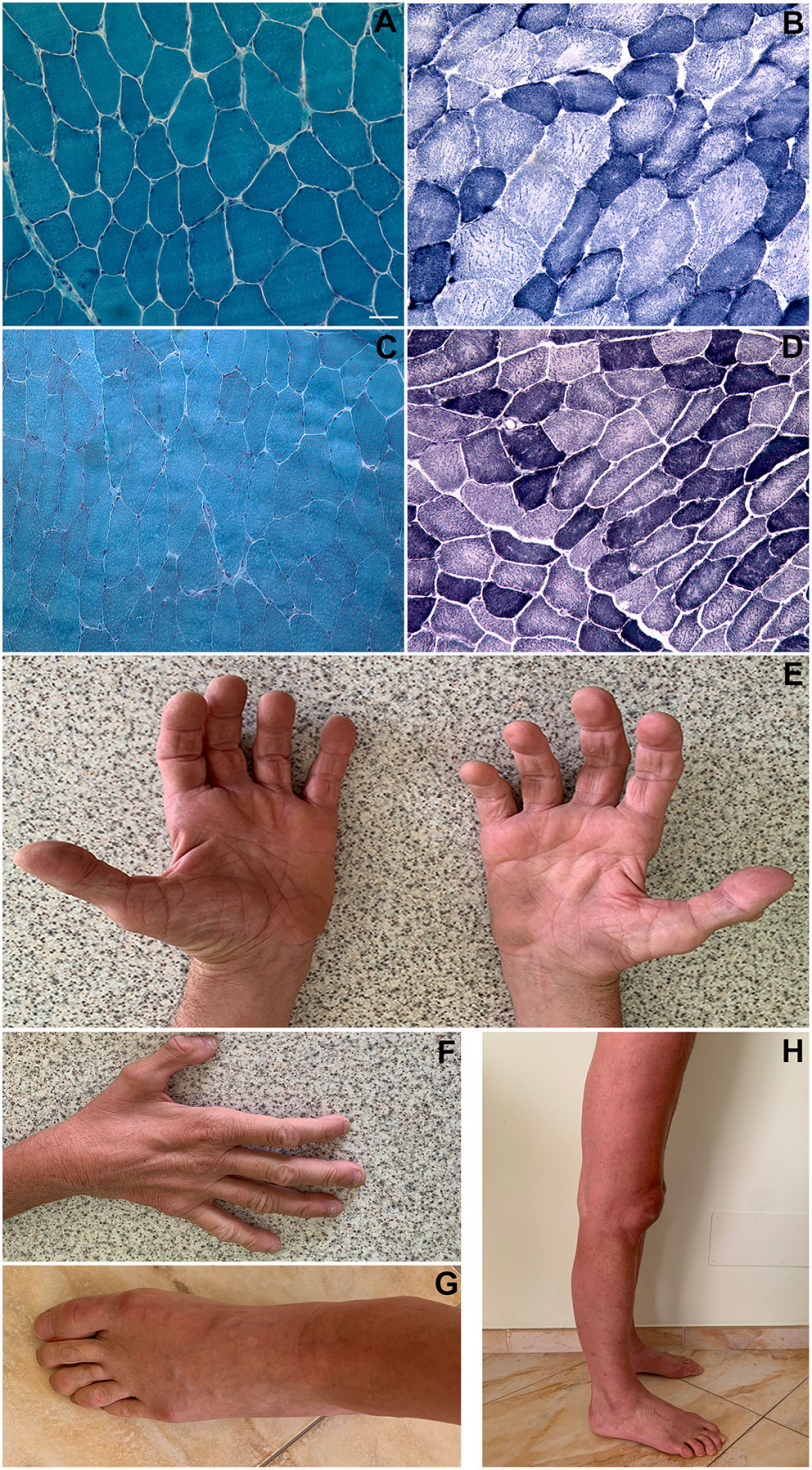
Histopathological findings. Patient 1 (biceps brachii muscle; magnification: 200x): Gomori trichrome stain showing a slightly increased variability in muscle fibers diameter; no rimmed vacuoles and no sarcoplasmic or intranuclear inclusions were present **(A)**. NADH-TR stain showing “moth-eaten” or “core-like” areas of oxidative enzyme activity reduction in both fiber types and increased subsarcolemmal activity in some type I fibers (darker blue areas) **(B)**. Patient 2 (vastus lateralis muscle; magnification: 200x): Gomori trichrome stain showing an almost normal fiber variability pattern; no rimmed vacuoles and no inclusions were present **(C)**. NADH-TR stain shows slightly “core-like” reduction of central oxidative enzyme activity in both fiber types **(D)**. Patient 1 pictures showing intrinsic hand, foot, leg and thigh muscles wasting **(E–H)**. Scale bar: 25 μm.

Neurological examination at the age of 39 revealed forearm pronators, finger flexors and intrinsic hand muscles moderate weakness and wasting with mild asymmetry, a selective moderate weakness of the ankle plantar flexors and posterior leg muscles wasting, while all other lower limbs muscles showed only mild strength reduction. As the disease was progressing, weakness and wasting became more widespread and involved more proximal muscles. Last clinical examination at the age of 52 showed a severe muscle atrophy of lower and upper limbs (Figure 1) with moderate-severe muscle weakness more evident at distal upper than lower limbs. Mild facial muscular weakness was evident together with mild dysphonia. The patient is still able to stand with support but not to walk.

No cardiac or respiratory involvement were present. Creatine kinase (CK) levels were only moderately increased (range along time from 300 to 850 UI/L, normal values: 24–195) and electromyography displayed chronic myopathic signs with distal neuropathic signs at upper and lower limbs muscles.

The patient performed three muscle biopsies along time: all of them revealed an increased variability in muscle fibers diameter, a small number of centralized nuclei, central areas with reduced oxidative enzyme activity in both fiber types and increased subsarcolemmal activity in some type I fibers (Figures 1A,B). Immunostaining of muscle membrane proteins (Dystrophin, Caveolin-3, sarcoglycans) was normal.

**Figure 2:**
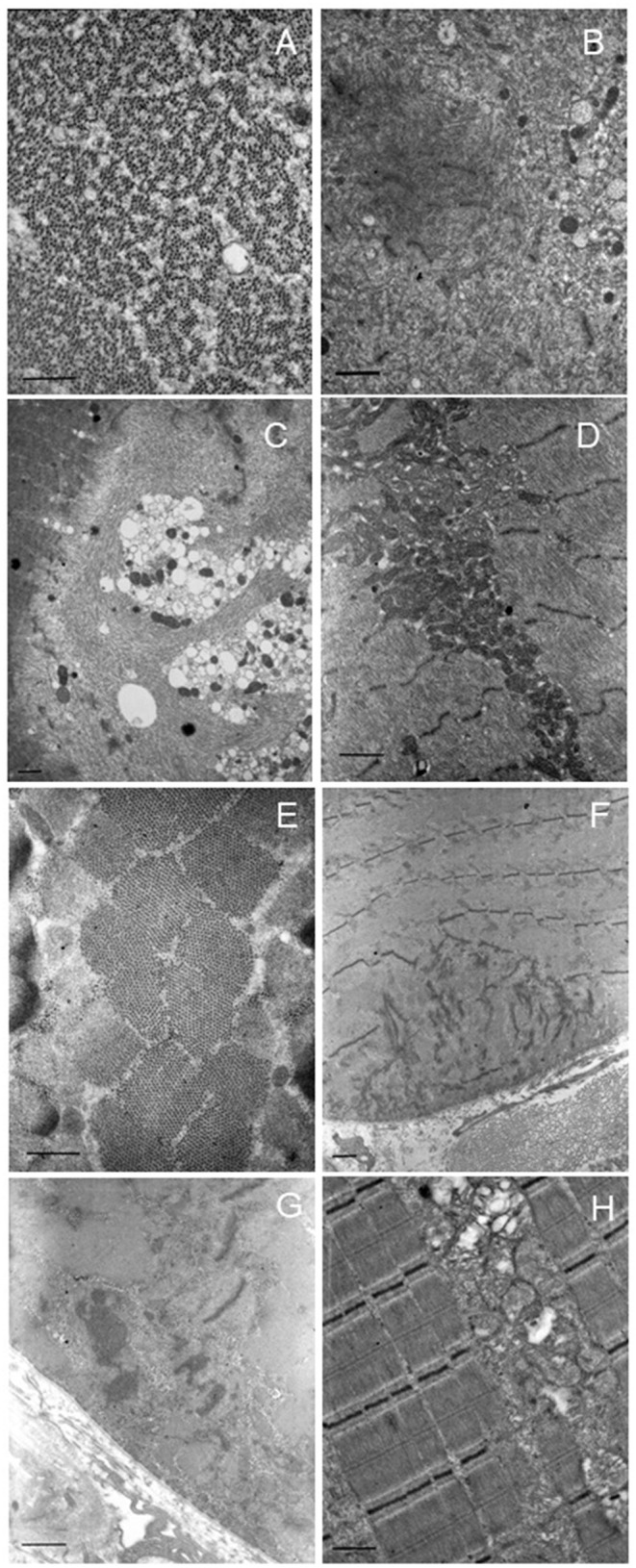
Electron microscopy. Representative images of patients 1 **(A–D)** and 2 **(E–H)**. Patient 1: altered ultrastructural sarcomere pattern in transverse section **(A)**. Degeneration of sarcomere structure with extended area of myofibrillar disorganization in the sarcoplasm **(B,C)**. Also, many cytoplasmic vacuoles **(C)**. Collection and increase of intermyofibrillar mitochondria with normal morphology **(D)**. Patient 2: normal appearance of sarcomere pattern **(E)**. Degeneration of sarcomere structure with extended area of myofibrillar disorganization at the sub-sarcolemma level **(F)**. Area of myofibrillar disorganization with cytoplasmic bodies **(G)**. Abnormal mitochondrial accumulation, some mitochondria are swollen or with altered cristae **(H)**. Scale bar: short dash 463 nm, long dash 926 nm.

Electron microscopy showed the presence of unstructured fibers, as the normal sarcomeric organization is no longer distinguishable (in some fields only a residual Z-disk was present) and small vacuoles with loose material inside, without evidence of collections of filamentous material; several mitochondria with altered or elongated shape, sometimes collected in small groups, and cytoplasmic dilations within several fibers were also noted (Figures 2A–D).

Muscle imaging through magnetic resonance of the lower limbs (performed at the age of 47) showed diffuse and severe muscle atrophy more evident in the posterior portion of the legs and signs of fatty substitution of the paravertebral muscles.

**Figure 3:**
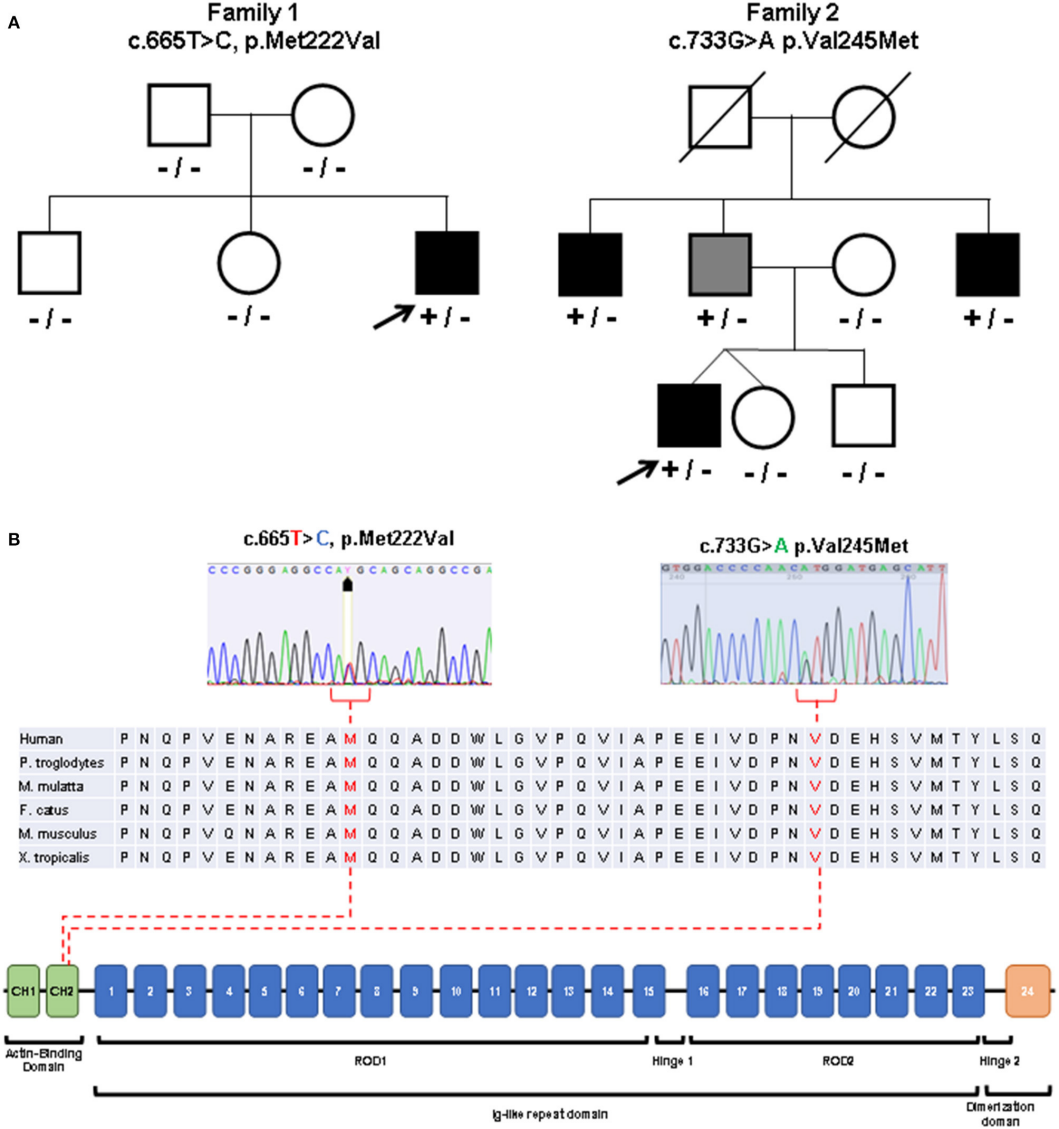
Molecular studies. **(A)** Pedigrees of the described families. Index patients are indicated with arrows. Black symbols indicate clinically affected subjects. Gray symbol indicates subclinical involvement. Available genotypes are reported under the corresponding subjects. [**(B)**, top] Variants identified in the patients (sequence electropherograms) affecting conserved amino acidic residues within the actin binding domain. [**(B)**, bottom] Diagram showing the structure of FLNC gene and the protein domains observed in the encoded protein (Filamin-c).

Sanger sequencing of genes encoding Calpain 3, Lamin A/C, Selenoprotein, Valosin Containing Protein (VCP), Fukutin related protein (FKRP), Dysferlin, Anoctamin 5, GNE, dynactin subunit 1 (DCTN1) and testing for Survival Motor Neuron (SMN1) deletions were negative. Exome sequencing performed on the Trio revealed a novel *de novo* heterozygous missense variant c.665T>C in the *FLNC* gene (NM_001458, exon 3) leading to p.Met222Thr substitution in the actin binding region of the Filamin c protein (Figure 3). No other suspicious variants were detected in other distal myopathy-related genes (and included in the relative curated gene list of Panel App).

The Met222 residue is evolutionary conserved from human to Xenopus and different prediction tools support a likely pathogenic effect of the identified variant (Supplementary Table 1). The patient's asymptomatic siblings tested negative for the *FLNC* variant.

### Patient 2

A 33-year-old man came to our attention with a clinical history of fatigue and progressive bilateral hypotrophy of the posterior thigh muscles and distal quadriceps, with associated foot plantar flexion weakness, lasting about 3 years. Currently, he is no longer able to lift himself on his toes and has suspended sports activity.

Electromyography showed predominantly myopathic changes in the proximal muscles of the four limbs (i.e., ileo-psoas, quadriceps femoris, deltoid) with additional aspects of active denervation in the triceps surae muscles (positive sharp waves, atypical repetitive discharges) and mixed neuropathic and myopathic features in the tibialis anterior.

Neurological examination revealed bilateral mild weakness of opponens pollicis and extensor digitorum and severe weakness of the gastrocnemius muscles, tibio-tarsal joint tension bilaterally, wasting of the posterior thigh and leg muscles. Creatine kinase levels were moderately elevated (652 UI/L). Echocardiography excluded structural cardiac alterations; there were no symptoms or signs of respiratory involvement.

The patient underwent open biopsy of the right vastus lateralis muscle. Histological examination showed mild muscular alterations, characterized by a diffuse reduction of oxidative enzyme activity with moth-eaten or core-like appearance (Figures 1C,D). Electron microscopy showed Z disk alterations (streaming or loss) in some fields, small subsarcolemmal or endomysial collections of mitochondria, in some cases of elongated shape (Figures 2E–H).

**Figure 4:**
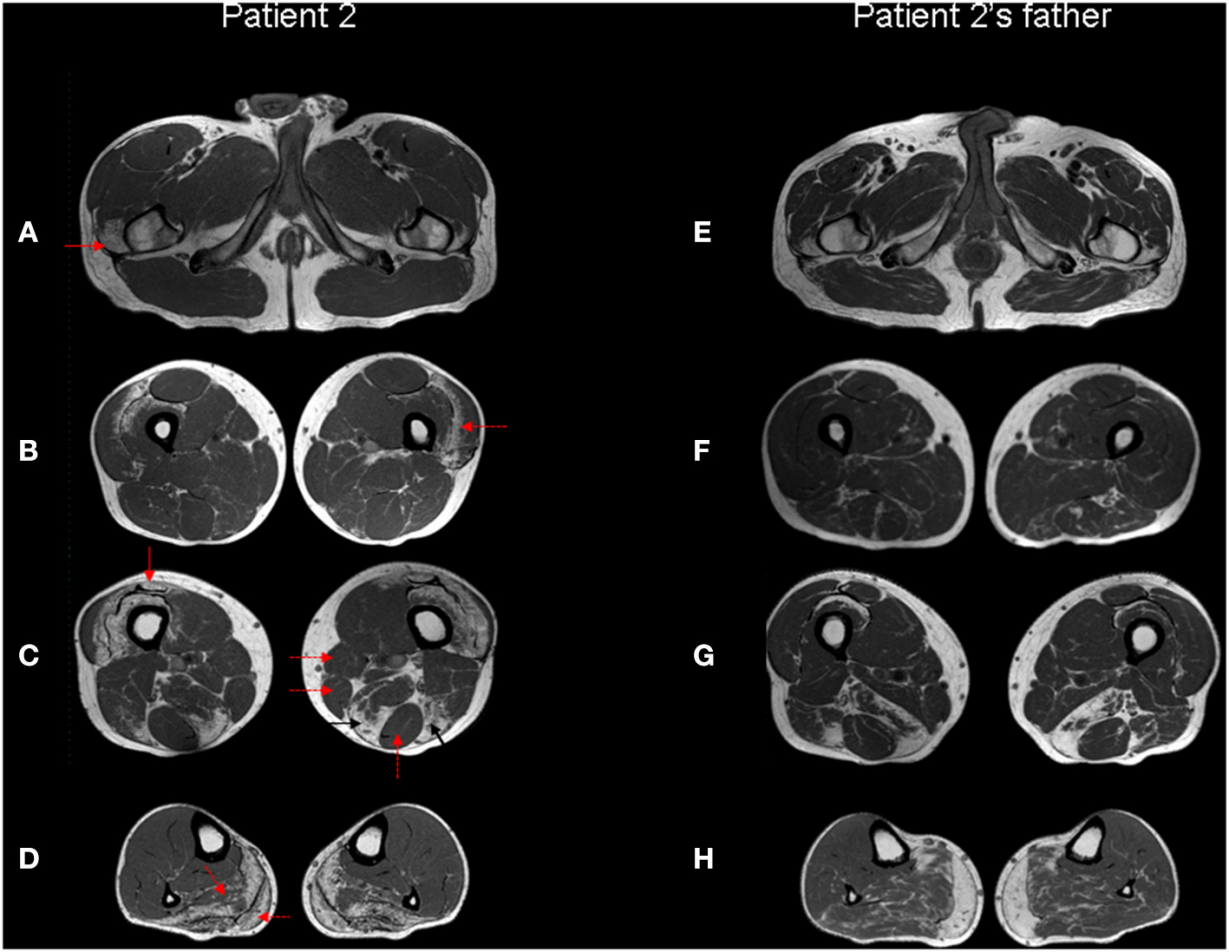
Muscle MRI. Patient 2 **(A–D)** and patient 2's father **(E–H)** Turbo Spin Echo (TSE) axial T1 MRI images from thigh **(A–C, E–G)** to leg **(D,H)**. The patient's images show bilateral symmetric selective fatty degeneration of the distal insertion of the gluteus medius muscle [**(A)**, red arrow], subfascial fatty degeneration of vastus lateralis and intermedius muscles [**(B)**, red dotted arrow], with a relative sparing of rectus femoris, involved only at the distal insertion [**(C)**, red arrow]; fatty degeneration and wasting of semimembranosus and long head of biceps femoris muscle [**(C)**, black arrows] with relative preservation of gracilis, sartorious and semitendinosus muscles [**(C)**, red dotted arrows]. In the lower leg, there is a predominant fibroadipose replacement of the posterior muscles, primarily affecting the soleus and the medial gastrocnemius muscles [**(D)**, red dotted arrows]. The same involvement pattern of selected muscle groups can also be observed in the patient's father, although it is less pronounced except at the level of the distal leg.

Lower limbs muscle MRI showed discrete muscle wasting of the posterior region of the lower legs and mild atrophy of the distal portion of quadriceps and hamstring muscles. Signs of adipose replacement are present in the same muscles, particularly in the leg (Figures 4A–D).

Patient 2 underwent NGS-based panel sequencing targeting main genes involved in muscular dystrophies and distal myopathies (Supplementary Table 2). NGS analysis disclosed the novel heterozygous *FLNC* actin-binding domain missense variant c.733G>A (exon 4, p.Val245Met) (Figure 3), predicted as pathogenic (Supplementary Table 1). The same variant was identified in the clinically asymptomatic 65-year-old patient's father who displayed normal CK levels (51 UI/L), mixed neurogenic and myopathic motor unit potentials at the level of the medial gastrocnemius muscle bilaterally, chronic myogenic potentials in the four limbs' proximal muscles, with normal nerve conduction velocities. Muscle MRI displayed the same pattern of adipose replacement, albeit to a lower degree, previously observed in Patient 2 (Figures 4E–H). The dizygotic twin sister and the brother of the patient tested negative for the *FLNC* variant. Finally, the c.733G>A change was detected in two paternal uncles. One of them is a 60 years old man who is unable to lift on his toes. His CK levels are modestly increased (215 UI/L) and his neurophysiologic examination shows chronic sensory-motor mixed neuropathic signs and mixed neurogenic and, especially, myopathic motor unit potentials in the distal legs muscles. The second paternal uncle, aged 57 years, is affected by dilated cardiomyopathy without apparent skeletal muscle involvement. He was tested on a gene panel containing 42 genes currently known to be associated with monogenetic cardiomyopathy (http://www.umcutrecht.nl/NGS; panel car01 version 21.1) which disclosed the same c.733G>A (p.Val245Met) *FLNC* variant. No other clinical information is available.

In silico prediction tools including CADD scores supported the pathogenic role of the variants identified in the patients (Supplementary Table 2).

## Discussion

In the present report we identified two novel *FLNC* variants affecting conserved residues in the ABD. The clinical, radiological and histological findings contribute to expand the heterogenous phenotypic manifestations and skeletal muscle involvement observed in patients with filaminopathies.

A distal myopathy phenotype has to date been associated with missense variants in the calponin homology 2 (CH2) domain of the ABD (5, 7) or frameshift variants in the immunoglobulin-like domain 15 of ROD1 domain of *FLNC* gene (8, 9).

Distal ABD-filaminopathy usually presents with handgrip weakness followed by posterior leg muscles involvement with slow clinical progression (5, 10). Gemelli and co-authors previously described a variant (c.664>G; p.M222V, the same residue found mutated in Patient 1) in two siblings presenting progressive distal myopathy starting in their third decade (7). Although presenting a similar age at onset, our case is characterized by an initial involvement of the upper limbs, an earlier involvement of the lower limbs, and raised CK levels. The clinical features of Patient 2 overlaps those described in other patients with ABD Filamin-c mutations, except for the higher CK levels (5). However, differently from previous reports, heterogenous presentations ranging from subclinical involvement of the distal leg muscles to apparently isolated cardiac involvement were observed in his family members harboring the same defect. Reduced penetrance and variable expressivity had been also previously described for *FLNC* variants associated with cardiac involvement (11, 12).

The histopathology of distal ABD-filaminopathy shows unspecific myopathic changes (10). In accordance with these data, and unlike the case already described in the literature (7), muscle biopsy findings in Patient 1 did not include myofibrillar aggregates nor intracytoplasmic or subsarcolemmal vacuoles. Likewise, immunohistochemical analysis of the Patient 2's muscle biopsy with antibodies directed against Desmin, Myotilin and αB-crystallin and ultrastructural analysis did not detect the characteristic protein aggregates of myofibrillar myopathies (1, 3).

Mixed myogenic and neurogenic pattern and spontaneous activity (positive sharp waves) in the anterior tibial muscle, with normal motor nerve conduction velocities, have already been described in Filamin C distal myopathy (8). An initial axonal motor neuropathy, with no significant abnormalities at the muscle needle exam, was suspected in another patient carrying a substitution in the Filamin C ABD causing a distal myofibrillar myopathy (7). Although in both our patients nerve conduction studies did not show reduced velocities and muscle biopsies did not reveal signs of neurogenic damage, concomitant neuropathy cannot be completely ruled out, as previously discussed (1).

The available muscle MRI data in filaminopathy show the following features: in the pelvis, initial changes are relatively mild. In the thigh, semimembranosus, adductor magnus and longus, long head of biceps femoris, vastus intermedius and medialis are most affected, whereas semitendinosus, rectus femoris, sartorius, and gracilis are relatively spared. In the lower leg, the soleus muscle shows pronounced fatty changes; tibialis anterior and medial gastrocnemius muscles are involved later, while peroneal muscles and lateral gastrocnemius are relatively spared even in more advanced disease (2, 13, 14). Although in Patient 1 the degree of fibroadipose replacement at the time of MRI is too advanced to recognize a peculiar pattern, this is certainly identifiable in Patient 2 and, more importantly, allows the identification of subclinical muscle involvement in his clinically asymptomatic father.

Our study contributes to expand the number of ABD variants of Filamin C associated with distal myopathy. Even in the absence of obvious alterations at standard microscopy, ultrastructural analysis might show the subversion of the intermyofibrillar network and mitochondrial alterations associated with myofibrillar myopathies (15, 16) but without protein aggregates. Muscle MRI is advised to recognize specific patterns of muscle involvement in affected patients and asymptomatic carriers.

## Data Availability Statement

The original contributions presented in the study are included in the article/Supplementary Material, further inquiries can be directed to the corresponding author/s.

## Ethics Statement

The studies involving human participants were reviewed and approved by Comitato Etico Milano Area 2 Fondazione IRCCS Ca' Granda Ospedale Maggiore Policlinico (Milan, Italy). The patients/participants provided their written informed consent to participate in this study. Written informed consent was obtained from the individual(s) for the publication of any potentially identifiable images or data included in this article.

## Author Contributions

DV and MD'A interpreted the results, conceived the idea, revised the literature, wrote the manuscript, and made the clinical evaluation. DR, AC, and EP performed genetic analysis. LN performed ultrastructural studies. CC performed muscle MRI studies. MM, DR, GC, and MB performed a critical revision of the manuscript for important intellectual content. All authors have read and approved the manuscript.

## Funding

This study was (partially) funded by Italian Ministry of Health—Current research IRCCS. The work was supported by funds from the Fondazione Regionale Lombarda per la Ricerca Biomedica (FRRB) grant Care4Neurorare (to MB). This work is generated within the European Reference Network for Neuromuscular Diseases.

## Conflict of Interest

The authors declare that the research was conducted in the absence of any commercial or financial relationships that could be construed as a potential conflict of interest.

## Publisher's Note

All claims expressed in this article are solely those of the authors and do not necessarily represent those of their affiliated organizations, or those of the publisher, the editors and the reviewers. Any product that may be evaluated in this article, or claim that may be made by its manufacturer, is not guaranteed or endorsed by the publisher.
