# Supplementary material for: Case Reports: Novel Missense Variants in the Filamin C Actin Binding Domain Cause Variable Phenotypes
Source: Front Neurol. 2022 Jul 12;13:930039. doi: 10.3389/fneur.2022.930039 (PMC9315448; doi:10.3389/fneur.2022.930039)
Supplement: Supplementary file 1 [file Data_Sheet_1.PDF]

Supplementary Figure 1 Timeline of relevant clinical signs and symptoms (blue squares), and of diagnostic assessments (red squares). OM: optic microscopy; EM: electron microscopy; MRI: magnetic resonance imaging; FLNC: Filamin C.

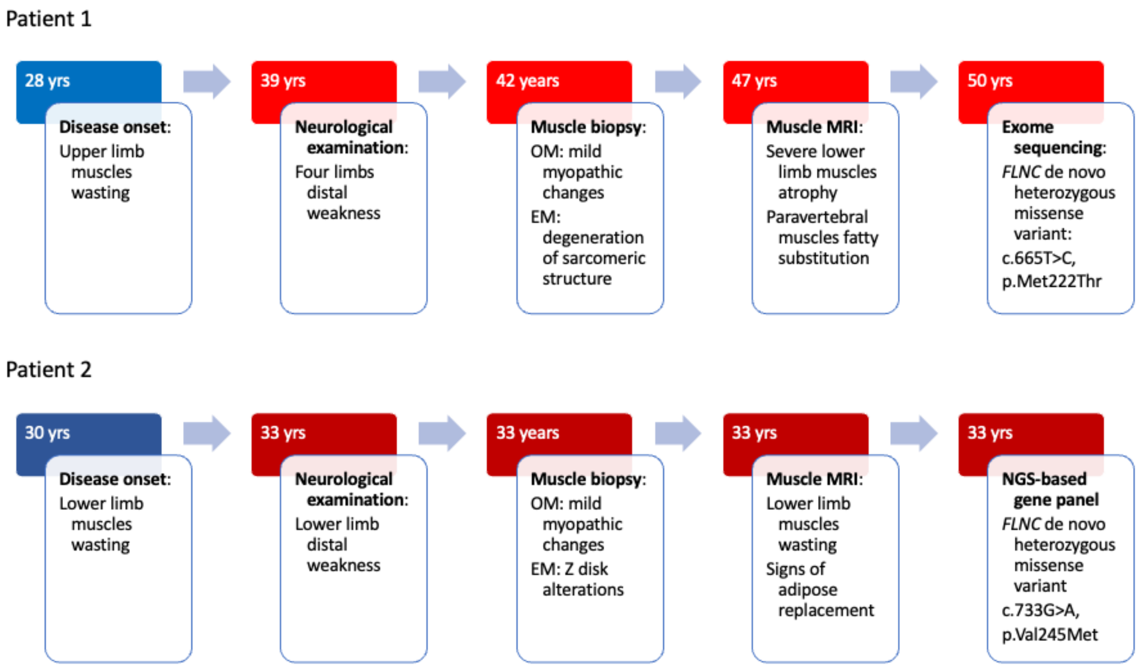

Supplementary Table 1 In silico analysis of pathogenicity of the identified variants (P = Pathogenic; LP: Likely pathogenic) according to different tools: CADD (https://cadd.gs.washington.edu), SIFT (https://sift.bii.a-star.edu.sg), PolyPhen-2 (http://genetics.bwh.harvard.edu/pph2/), Mutation Assessor (http://mutationassessor.org/r3/) and the criteria of pathogenicity according to the guidelines of the American College of Medical Genetics (ACMG).

**Coordinates**	**cDNA (NM_001127487)**	**AA change**	**CADD**	**SIFT**	**Polyphen2**	**Mutation Assessor**	**ACGM**
7:128837223T/C	c.665T>C	p.Met222Thr	27	P	P	P	LP (PM2, PM1, PP3)
7:128477485G/A	c.733G>A	p.Val245Met	28	P	P	P	LP (PM2, PM1, PP3)

Supplementary Table 2 Genes included in the NGS-panel which was used for Patient 2 diagnosis.

Gene	Inheritance	OMIM	Locus
AGL	Ar	232400, 232400	*1p21.2*
AMPD1	Ar	615511	*1p13.2*
ANO5	AD, Ar	166260, 613319, 611307	*11p14.3*
B3GALNT2	Ar	615181	*1q42.3*
B4GAT1	Ar	615287	*11q13.2*
BVES	Ar	616812	*6q21*
CACNA1S	AD	170400, 601887, 188580	*1q32.1*
CAPN3	AD, Ar	618129, 253600	*15q15.1*
CAV3	AD, DD	192600, 123320, 611818, 614321, 606072	*3p25.3*
CLCN1	AD, Ar	160800, 255700	*7q34*
COL6A1	AD, Ar	158810, 254090	*21q22.3*
COL6A2	Ar, AD	255600, 158810, 254090	*21q22.3*
CPT2	Ar, AD	600649, 608836, 255110, 614212	*1p32.3*
CRPPA	Ar	614643, 616052	*7p21.2*
DAG1	Ar	616538, 613818	*3p21.31*
DES	AD, Ar	604765, 601419, 181400	*2q35*
DMD	XLr	300376, 302045, 310200	*Xp21.2-p21.1*
DNAJB6	AD	603511	*7q36.3*
DPM3	Ar	618992, 618992	*1q22*
DYSF	Ar	254130, 253601, 606768	*2p13.2*
FKRP	Ar	613153, 606612, 607155	*19q13.32*
FKTN	Ar	611615, 253800, 613152, 611588	*9q31.2*
FLNC	AD	617047, 617047, 614065, 609524	*7q32.1*
GAA	Ar	232300	*17q25.3*
GBE1	Ar	232500, 263570	*3p12.2*
GFPT1	Ar	610542	*2p13.3*
GMPPB	Ar	615350, 615351, 615352	*3p21.31*
GNE	Ar, AD	605820, 269921	*9p13.3*
GTDC2	Ar	614830	*3p22.1*
HNRPDL	AD	609115	*4q21.22*
ISPD	Ar	614643	*7p21.2*
KCNJ2	AD	170390, 613980, 609622	*17q24.3*
LAMA2	Ar	607855, 618138	*6q22.33*
LARGE1	Ar	613154, 608840	*22q12.3*
LIMS2	AD	613980	*2q14.3*
LMNA	AD, Ar	115200, 605588, 181350, 616516, 610140, 176670, 151660, 212112, 248370, 613205, 619793	*1q22*
MYOF	AD	619366	*10q23.33*
MYOT	AD	609200, 182920	*5q31.2*
PLEC	Ar, AD	616487, 131950, 226670, 612138, 613723	*8q24.3*
POGLUT1	Ar, AD	617232, 615696	*3q13.33*
POMGNT1	Ar	253280, 613151, 613157, 617123	*1p34.1*
POMGNT2	Ar	614830, 618135	*3p22.1*
POMK	Ar	616094, 615249	*8p11.21*
POMT1	Ar	236670, 613155, 609308	*9q34.13*
POMT2	Ar	613150, 613156, 613158	*14q24.3*
POPDC3	Ar	618848	*6q21*
PYGM	Ar	232600	*11q13.1*
PYROXD1	Ar	617258	*12p12.1*
RXYLT1	Ar	615041	*12q14.2*
RYR1	AD, Ar	117000, 619542, 255320, 145600	*19q13.2*
SCN4A	AD, Ar	170500, 613345, 614198, 608390, 168300	*17q23.3*
SGCA	Ar	608099	*17q21.33*
SGCB	Ar	604286,	*4q12*
SGCD	Ar	606685, 601287	*5q33.2-q33.3*
SGCG	Ar	253700	*13q12.12*
SMPX	XLD, XLR	300066, 301075	*Xp22.12*
TCAP	AD, Ar	607487, 601954	*17q12*
TMEM5	Ar	615041	*12q14.2*
TNPO3	AD	608423	*7q32.1*
TOR1AIP1	Ar	617072	*1q25.2*
TRAPPC11	Ar	615356	*4q35.1*
TRIM32	Ar	615988, 254110	*9q33.1*
